# The Financial and Workforce Impact of Medication Errors in the Finnish Public Healthcare System: A Pilot Study

**DOI:** 10.1177/11786329261427522

**Published:** 2026-02-23

**Authors:** Jonna-Carita Kanninen, Raimo Ojala, Jouni Ahonen, Hannu Kautiainen, Anu Holm, Ville Valkonen

**Affiliations:** 1 Wellbeing Services County of North Savo, Hospital Pharmacy, Kuopio, Finland; 2 Primary Health Care Unit, Kuopio University Hospital, Finland; 3 Folkhälsan Research Center, Helsinki, Finland; 4 Faculty of Medicine, University of Turku, Finland; 5 School of Pharmacy, University of Eastern Finland, Kuopio, Finland

**Keywords:** medication errors, adverse events, healthcare costs, resource allocation, patient safety

## Abstract

**Background:**

Medication errors (MEs) pose a significant challenge to patient safety and healthcare efficiency. In addition to clinical consequences, MEs contribute to increased healthcare expenditures and resource utilization. However, comprehensive cost assessments of MEs, including staff time and service costs, remain limited.

**Objectives:**

The aim of this pilot study is to examine the costs associated with MEs regionally and nationally in Finland.

**Design:**

Cross-sectional study.

**Methods:**

This study assessed the financial and resource burden of MEs in the Finnish public healthcare system using a survey conducted in the wellbeing services county of North Savo in Eastern Finland. The study perspective is economic and healthcare system-focused. Healthcare professionals in charge of patient safety reporting estimated the workforce impact of MEs by assessing the time spent, the corrective measures taken, and the additional interventions required to manage these events. Cost calculations were based on salary data from the 2023 financial administration statistics and service costs from the Finnish Institute for Health and Welfare (THL) database, adjusted to 2023 monetary value. An estimator, derived from regional data, was applied to extrapolate the nationwide economic burden of MEs.

**Results:**

Medication errors (MEs) impose a substantial financial burden, with an estimated mean cost of €138 per event and a total annual cost of €686 274 in the wellbeing services county of North Savo. Extrapolated to the national level, the annual impact was estimated at €15.5 million. The main cost drivers were the work time expenditures of nurses and physicians involved in managing these events.

**Conclusion:**

Managing MEs demands extra work of healthcare personnel, which is a considerable waste of resources. Most of the events are preventable. That is why effective safety strategies are needed, and prompt action taken to improve quality of care and reduce unnecessary costs.

## Introduction

Medication errors (MEs) are a major global health concern, causing significant patient harm and placing a substantial financial burden on healthcare systems.^1-3^ An ME is defined as any preventable event that may cause or lead to inappropriate medication use or patient harm while the medication is in the control of the healthcare professional, patient, or consumer.^4^ These errors can occur at any stage of the medication process, including prescribing, preparation, distribution, administration, or monitoring.^5^

In the United States alone, adverse events (AEs) in healthcare, including MEs, are estimated to contribute to up to 98 000 deaths annually,^6^ ranking them the third leading cause of death after heart disease and cancer.^7^ In addition, approximately 1.3 million individuals are harmed by MEs each year.^8^ Beyond their clinical consequences, MEs also represent a considerable economic burden. Globally, the cost associated with MEs is estimated at $42 billion USD per year.^9^ More recent studies suggest that MEs are the most common preventable cause of adverse drug events (ADE),^10^ with cost exceeding $54 billion USD in OECD countries.^11^ In Finland, the National Audit Office has estimated that quality deviations, including MEs and other AEs, result in annual healthcare costs exceeding one billion euros.^12^ ADEs are also estimated to account for approximately one quarter of emergency department visits by elderly patients.^13^ However, there is currently no national-level evaluation of healthcare costs specifically attributable to MEs in Finland.

Estimating the financial burden of MEs is challenging due to variations in cost assessment methods and perspectives (eg, service providers, patients, or third-party payers) across studies.^14-16^ While previous studies have examined the economic impact of MEs,^17-20^ they do not consider the cost of staff time spent managing these errors. In addition, no standardized approach exists for calculating their total economic impact.^21^ The aim of this pilot study is to examine the costs associated with MEs regionally and nationally in the Finnish public healthcare system by assessing the working time of healthcare personnel and other financial resources required to manage these errors and implement corrective measures.

## Materials and Methods

This study was conducted as a cross-sectional study in within the wellbeing services county of North Savo. The study perspective is economic and healthcare system-focused, specifically from the viewpoint of the Finnish public healthcare system. The wellbeing services county of North Savo, established in 2023, is one of Finland’s 21 public regional social and healthcare service providers^22,23^ covering both specialized medical care and primary healthcare services. North Savo County, located in Eastern Finland, has a population of approximately 248 000 residents.^24^

### Survey Design

The study consisted of an electronic survey (Surveypal©) aimed at assessing resource utilization and costs associated with MEs within the wellbeing services county of North Savo. The survey targeted healthcare professionals responsible for handling safety incident reports using HaiPro^®^,^25^ a web-based tool for reporting patient safety incidents. The survey was conducted between 21 May, 2024, and 30 June, 2024. Completing the survey took approximately 5 to 10 minutes, and responses were submitted anonymously. A link to the online survey was distributed via email to all departmental managers (n = 1004) and medication contact persons in the departments (n = 481) within the wellbeing services county. The survey distribution covered both specialized medical care and primary healthcare services within the wellbeing services county, including all clinical specialties. The survey was also permitted and requested to be forwarded to relevant staff.

The survey collected work time usage, corrective actions, and potential improvements in medication safety. Respondents were instructed to report only additional services directly caused by the ME, not routine patient care. Costs were thus attributed only to corrective or extra interventions due to MEs. The survey was developed by the researchers and was piloted in this study. The survey consisted of the following five key components:

(i) *Type of the most recent MEs* (eg, prescribing error, administration error, dispensing error, documentation error, monitoring error, storage error, or unexpected patient reaction),
(ii) *Error type* – whether the event resulted in actual patient harm or was a near-miss,
(iii) *Work time estimation* – estimated time (in minutes) spent by different healthcare professionals (eg, practical nurses, nurses, physicians, pharmacists, and other professionals) on detecting, investigating, correcting, and managing the error,
(iv) *Additional interventions required* – whether correcting the incident required additional actions beyond routine duties. If additional actions were required, participants specified the type of intervention (eg, patient information, clinical procedures, patient monitoring, hospital admission, ambulance transport, or intensive care), and
(v) *Proposals for improving medication safety* – respondents were asked to evaluate which measures could improve medication safety in their workplace (eg, enhanced guidelines, additional training, process improvements, better communication, or optimized use of HaiPro reporting). An open-ended section allowed participants to provide additional suggestions on how to improve medication safety in their unit.

Inclusion criteria for the survey comprised healthcare professionals involved in ME reporting within the wellbeing services county of North Savo. Exclusion criteria included responses unrelated to MEs or submitted outside the study period.

### Work Time and Service Costs

Work time costs were calculated using the average salaries of various healthcare professionals based on the 2023 financial administration statistics of the wellbeing services county of North Savo (Table 1). In the survey, respondents were asked to describe the events in more detail, and these events were subsequently classified into different service categories by three researchers. All classifications were reviewed collectively to achieve consensus. In addition, the frequency and type of MEs were analyzed based on the survey responses.

**Table 1.** Work Time Cost Per Medication Error by Healthcare Professional Reported in a Survey in the Wellbeing Services County of North Savo in 2024 (n = 130).

Healthcare professional	Number n (%)	Mean time usage per case (h) Mean (SD) [range]	Unit cost* (€/h)	Cost per error (€) Mean (95% CI)
Practical nurse	38 (29)	0.10 (0.26) [0.0-2.0]	16.0	1.7 (1.1-2.5)
Nurse	97 (75)	0.89 (6.30) [0.0-72.0]	18.5	16.4 (5.3-16.1)
Physician	44 (34)	0.10 (0.21) [0.0-1.0]	34.9	3.4 (2.4-6.1)
Pharmacist	11 (8)	0.08 (0.37) [0.0-3.0]	23.2	1.8 (0.7-3.6)
Other	28 (22)	0.07 (0.21) [0.0-1.5]	20.3	1.5 (0.8-2.3)
Sum	NA	1.24 (7.68) [0.0-72]	112.9	24.8 (4.39-44.21)

* The average salaries of various healthcare professionals based on the 2023 financial administration statistics of the wellbeing services county of North Savo.

The cost calculations for services were based on the National Institute for Health and Welfare report on unit costs in Finnish health and social care in 2017.^26^ The 2017 prices for each procedure and service were corrected to reflect 2023 monetary value using Statistics Finland’s consumer price index^27^ (Table 1). The average cost of a single medication dose was calculated using the total cost of all dispensed medications in 2023 from the hospital pharmacy of the wellbeing services county of North Savo, divided by the total number of dispensed units. This resulted in an average medication dose price of €2.88.

### Statistical Analysis

The statistics values were presented as means with standard deviation (SD) or counts with percentages (%). As the data for costs were highly skewed, bias corrected and accelerated bootstrap estimation (10 000 replications) was used to derive 95% confidence intervals. Incidence rates (per 1000 person years) with 95% confidence intervals (CI) were calculated assuming a Poisson distribution.

The regional ME incidence rate and mean cost per event were extrapolated to national population data using Statistics Finland’s population estimates for 2023. Stata 18.0 (StataCorp LP, College Station, TX, USA) was used for the statistical analyses.

## Results

Among the 130 reported MEs, 86 cases (66%) resulted in direct patient or customer harm, while 44 cases (34%) were near-misses.

**Figure 1.**
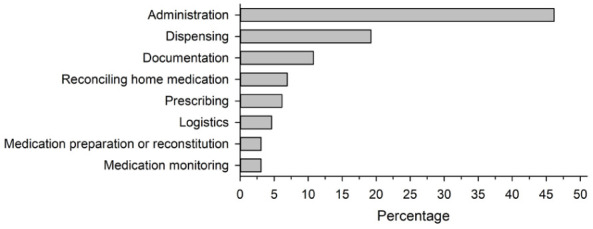
The types of medication errors reported in a survey in the wellbeing services county of North Savo in 2024 (n = 130). Logistics errors include the following types of errors: delivery error, storage error, and ordering error.

Administration errors were the most frequently reported, accounting for nearly 50% of cases. Dispensing errors were the second most frequent, followed by documentation errors. Other less common errors included logistical issues, errors in verifying home medication, prescribing errors, and monitoring errors (Figure 1).

### Work Time and Service Costs

The total work time per event was estimated at 1.24 hours (SD 6.31) [Range: 0.0-72.0]. The mean work time cost per event was €24.9 (95% CI: 13.0-55.1). Work time and cost per event by healthcare professional are presented in Table 1.

The number of service events reported in the MEs in the survey was 27, and their total cost was €14 635 (Table 2). The service costs per event (n = 130) was €114 (95% CI: 5-333). In cases where the event directly affected the patient or customer (n = 86), the service cost was €171 (95% CI: 8-556). Of the 86 cases affecting the patient or customer, 15 (17%) required a service event, while 71 (83%) did not.

**Table 2.** Service Events and Their Costs Relating to Medication Errors Reported in a Survey in the Wellbeing Services County of North Savo in 2024 (n = 130).

Service event	Number (n)	Service event cost (€)	Total (€)
Laboratory test	4	1.43*	5.72
Inpatient ward	3	4485.62*	13 456.86
Additional monitoring	11	41.69*	458.59
Home visit, nurse	1	98.87*	98.87
Gastrointestinal endoscopy	1	595.03*	595.03
Medication cost (1 unit, avg.)	7	2.88**	20.16
Sum	27	NA	14 635.23

* Based on the National Institute for Health and Welfare report on unit costs in Finnish health and social care in 2017^26^ and prices were adjusted to reflect 2023 monetary value using Statistics Finland’s inflation calculator.^27^

** Total cost of all dispensed medications in 2023 from the hospital pharmacy of wellbeing services county of North Savo divided by the total number of dispensed units.

**Figure 2.**
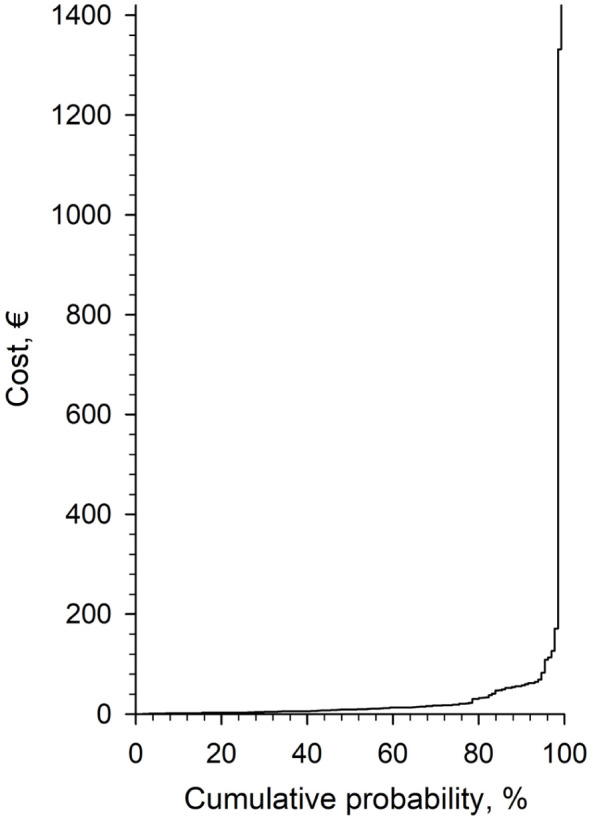
Distribution of service costs related to medication errors (n = 130) reported in a survey in the wellbeing services county of North Savo in 2024.

Figure 2 presents the cumulative distribution of medication error-related service costs. The majority of cases had relatively low costs, remaining below €200, while a small proportion of incidents resulted in significantly higher costs, exceeding €1000. The steep increase in the upper tail of the curve demonstrates that a few high-cost incidents contribute disproportionately to the total financial burden of MEs.

### Cost Estimation and Nationwide Extrapolation

The average cost per MEs, including work time and service costs, was estimated at €138 (95% CI: 22-373). Total number of MEs was 4973 in the wellbeing service county of North Savo in 2023 and estimated ME annual incidence was 20.1 per 1000 person-years (95% CI: 19.5-20.6). Using the annual estimator, the total annual cost of MEs in North Savo was €686 274 (including work time and service costs). In addition, in a nationwide extrapolation annual cost of MEs totaling €15 456 000 in Finland.

## Discussion

This pilot study provided a comprehensive assessment of the time and financial costs associated with MEs in the wellbeing services county of North Savo and produced a national-level estimate of their economic burden in the Finnish public healthcare system. The results indicated that MEs consume significant healthcare resources, including staff time, service costs, and additional corrective interventions. These findings align with previous studies highlighting the clinical and economic impact of MEs on healthcare systems globally.^1-3^

Unlike adverse drug reactions (ADRs), which are caused by the pharmacological properties of medications and are often unavoidable, MEs results from errors in the medication process.^28^ As MEs are considered preventable,^5^ they represent a highly relevant target for safety interventions.^9,29,30^ Preventing, mitigating, and addressing MEs, healthcare systems can reduce both unnecessary patient harm and the inefficient use of resources.^31^ In contrast to ADRs, which may occur despite correct and appropriate medication use, MEs represent a clear opportunity for both clinical and economic improvement.

Our findings confirmed that MEs place a significant financial burden on public healthcare systems. Both work time and service costs contribute notably to this burden. Nurses and physicians were identified as the professionals most involved in managing MEs, increasing resource use. A notable proportion of errors required additional interventions, which further amplified the total cost. These findings align with previous studies demonstrating the substantial costs associated with MEs and its economic implications.^17-19^

When extrapolated to a national level, the findings suggest that MEs contribute considerably to annual public healthcare expenditures across Finnish wellbeing service counties and regions. However, regional differences in population structure and healthcare utilization patterns, may introduce some bias. Also, differences in medication safety practices and incident reporting systems lead to fragmented data, which is not currently collected in a centralized manner. Similar challenges have been observed internationally, highlighting the need for standardized methodologies in both ME cost estimation and prevention.^14,20,21^ The cost estimates reported in this study are in line with those observed across Europe and globally, where MEs are identified as a major contributor to healthcare expenditures.^15,16^ Prior research has estimated that MEs cost European and worldwide healthcare systems billions of euros annually.^9,10,21^ This study adds nationally specific data from Finland to this discussion, reinforcing the importance of systematic cost assessments and the need for improved medication safety strategies and actions.^32^

Most reported MEs in this study resulted in direct harm to the patient or customer. However, near-miss events are also relatively common and are known to increase the risk of future AEs.^33^ To improve quality of care, it is essential to document near-misses, as they provide critical learning opportunities for improving error prevention and safety.^34^ It has been identified that a positive work environment is associated with higher rates of near-miss reporting,^35^ and reporting these events is a crucial first step toward preventing future MEs.^36^ In addition, clear and standardized policies and procedures are likely to increase the reporting rates.^36^ It is important to note that due to limitation of the data available in this pilot study, it evaluated only direct work time and service costs, not indirect costs, such as productivity loss, litigation, or longer-term morbidity. This is most likely leading to underestimation of total cost related to MEs. There is no standardized methodology for calculating ME costs that incorporates both direct and indirect costs.^21^ Comprehensive models, real-world data, and predictive tools are essential for more accurate cost assessments.

The most frequently reported errors were related to medication administration, followed by dispensing and documentation errors. These findings are consistent with previous research, which highlights administration-related MEs as a primary area of concern in clinical practice.^4,5^ Errors in verifying home medication, prescribing, and monitoring occurred less frequently but were still present. However, it is possible that these types of errors were underrecognized. International studies indicate that prescribing errors are actually the most prevalent,^37,38^ suggesting a need to enhance medication safety strategies particularly in underreported error categories. Even when patient harm does not occur, MEs result in additional costs due to detection, correction, and reporting processes, which all require substantial staff time and resources.^18^ However, the average costs associated with near-miss MEs are relatively lower than those that reach the patient, highlighting the importance of learning from near-miss errors and targeting improvement strategies to them as well, rather than focusing only on MEs with serious consequences. Even if the ME was a near-miss and was detected before it reached the patient, it is possible and quite likely that similar errors next time could result in patient harm and more costly corrective actions. This is consistent with the principle that MEs should always be aimed at preventing errors, making them detectable so that they can be intercepted, and providing means of mitigating them if they are not caught.^39^ Improvement measures are more effective and sustainable when they focus on systems, such as process improvements and fail-safes, rather than person-focused efforts that rely on individual memory and behaviour.^40,41^

To our knowledge, this is the first study to assess the economic burden of MEs in Finland. It provides real-world data on the time use and costs, providing valuable insights for national-level healthcare decision-making. In addition, the study includes data from both specialized and primary care, as wellbeing services counties are responsible for both sectors.

While the study provides valuable findings, some limitations should be acknowledged. First, the study data is limited only to public sector, due to the complexity of the healthcare delivery model in Finland, which involves the public, private and occupational sectors. Second, we do not know the total number of MEs during the study period or how many errors were left undocumented in reality. Also, the data were based on self-reported estimates of time and interventions, which may introduce recall bias. Differences in ME reporting practices across regions may also influence the accuracy of cost estimates. In addition, the study did not account for indirect costs, such as long-term patient health outcomes, legal expenses, or productivity losses, meaning the true economic burden is likely underestimated. Despite these limitations, the findings highlight the need for improved medication safety interventions and cost-effective strategies to reduce the impact of MEs on healthcare systems.

This pilot study provided a preliminary analysis of economic burden in the public healthcare system in Finland. Future research should build on these findings by incorporating data from multiple wellbeing services counties to enhance representativeness and generalisability. Importantly, the private and occupational healthcare sectors, both of which play a significant role in Finnish healthcare, should be included to capture the full scope of medication errors and related costs. Ideally, future studies should adopt a prospective design and utilize objective data sources such as electronic health records, incident reports, or administrative registers to improve data reliability and reduce bias. This study piloted the use of survey instrument to gather information of workload and service use relating to MEs providing useful information and study data. However, the survey could benefit from further testing and validation.

Future research should also assess the long-term cost-effectiveness of such programs and investigate how digital tools could help reduce ME-related costs in Finnish healthcare settings. Furthermore, artificial intelligence (AI) could support cost analysis and estimation of MEs, providing valuable insights for cost-effective interventions. Further studies are also needed on how AI can be integrated into existing healthcare systems, including ME reporting, and to support the identification of risks and errors in the medication process and to promote corrective and preventive actions.

## Conclusion

MEs impose a significant financial burden in public healthcare system in Finland, with an average cost of €138 per event and an estimated €15 million annually. Managing MEs demands extra work of healthcare personnel, which is a considerable waste of resources. Most of the events are preventable. That is why effective safety strategies are needed, and prompt action taken to improve quality of care and reduce unnecessary costs.

## References

[1] Adie K Fois RA McLachlan AJ Walpola RL Chen TF. The nature, severity, and causes of medication incidents from an Australian community pharmacy incident reporting system: the QUMwatch study. Br J Clin Pharmacol. 2021;87(12):4809-4822. 34022060 10.1111/bcp.14924

[2] Panagioti M Khan K Keers RN , et al. Prevalence, severity, and nature of preventable patient harm across medical care settings: systematic review and meta-analysis. BMJ. 2019;366:l4185. 10.1136/bmj.l4185 PMC6939648 31315828

[3] Coelho F Furtado L Mendonça N , et al. Predisposing factors to medication errors by nurses and prevention strategies: a scoping review of recent literature. Nurs Rep. 2024;14(3):1553-1569. 39051353 10.3390/nursrep14030117 PMC11270417

[4] National Coordinating Council for Medication Error Reporting and Prevention. About medication errors. What is a medication error? National Coordinating Council for Medication Error Reporting and Prevention; 2019. Accessed February 6, 2025. http://www.nccmerp.org/about-medication-errors

[5] Aronson JK. Medication errors: definitions and classification. Br J Clin Pharmacol. 2009;67(6):599-604. 19594526 10.1111/j.1365-2125.2009.03415.x PMC2723196

[6] Stelfox HT Palmisani S Scurlock C Orav EJ Bates DW. The “to err is human” report and the patient safety literature. Qual Saf Health Care. 2006;15(3):174-178. 16751466 10.1136/qshc.2006.017947 PMC2464859

[7] Makary MA Daniel M. Medical error—the third leading cause of death in the US. BMJ. 2016;353:i2139. 10.1136/bmj.i2139 27143499

[8] Wittich CM Burkle CM Lanier WL. Medication errors: an overview for clinicians. Mayo Clin Proc. 2014;89(8):1116-1125. 24981217 10.1016/j.mayocp.2014.05.007

[9] World Health Organization. Medication without harm. 2017. Accessed February 6, 2025. https://www.who.int/initiatives/medication-without-harm

[10] World Health Organization. Reporting and learning systems for medication errors: the role of pharmacovigilance centres. 2014. Accessed February 6, 2025. https://www.who.int/publications/i/item/9789241507943

[11] de Bienassis K Esmail L Lopert R Klazinga N. , The economics of medication safety: improving medication safety through collective, real-time learning. OECD Health Working Papers, No. 147, OECD Publishing, Paris, 2022. 10.1787/9a933261-en

[12] National Audit Office of Finland. Steering and Monitoring of Patient and Client Safety: Performance Audit Report (Audit Reports 7/2021). 2021. Accessed February 17, 2025. https://www.vtv.fi/app/uploads/2021/06/VTV-Tarkastus-7-2021-Potilas-ja-asiakasturvallisuuden-ohjaus-ja-seuranta.pdf

[13] Laatikainen O Sneck S Bloigu R Lahtinen M Lauri T Turpeinen M. Hospitalizations due to adverse drug events in the elderly: a retrospective register study. Front Pharmacol. 2016;7:358. 27761112 10.3389/fphar.2016.00358 PMC5051318

[14] Kirwan G O’Leary A Walsh C , et al. Potential costs and consequences associated with medication error at hospital discharge: an expert judgement study. Eur J Hosp Pharm. 2023;30(2):86-91. 35145001 10.1136/ejhpharm-2021-002697 PMC9986922

[15] Robinson EG Hedna K Hakkarainen KM Gyllensten H. Healthcare costs of adverse drug reactions and potentially inappropriate prescribing in older adults: a population-based study. BMJ Open. 2022;12(9):e062589. 10.1136/bmjopen-2022-062589 PMC9511550 36153031

[16] Arora V Moriates C Shah N. The challenge of understanding health care costs and charges. AMA J Ethics. 2015;17(11):1046-1052. 26595246 10.1001/journalofethics.2015.17.11.stas1-1511

[17] Gharekhani A Kanani N Khalili H Dashti-Khavidaki S. Frequency, types, and direct related costs of medication errors in an academic nephrology ward in Iran. Ren Fail. 2014;36(8):1268-1272. 24987790 10.3109/0886022X.2014.934650

[18] Choi I Lee SM Flynn L , et al. Incidence and treatment costs attributable to medication errors in hospitalized patients. Res Social Adm Pharm. 2016;12(3):428-437. 26361821 10.1016/j.sapharm.2015.08.006

[19] Boostani K Noshad H Farnood F , et al. Detection and management of common medication errors in internal medicine wards: impact on medication costs and patient care. Adv Pharm Bull. 2019;9(1):174-179. 31011571 10.15171/apb.2019.020 PMC6468220

[20] Rozenblum R Rodriguez-Monguio R Volk LA , et al. Using a machine learning system to identify and prevent medication prescribing errors: a clinical and cost analysis evaluation. Jt Comm J Qual Patient Saf. 2020;46(1):3-10. 31786147 10.1016/j.jcjq.2019.09.008

[21] Ranasinghe S Nadeshkumar A Senadheera S Samaranayake N. Calculating the cost of medication errors: a systematic review of approaches and cost variables. BMJ Open Qual. 2024;13(2):e002570. 10.1136/bmjoq-2023-002570 PMC11029430 38626938

[22] Ministry of Social Affairs and Health. Social and health services. 2021a. Accessed August 24, 2021. http://stm.fi/en/social-and-health-services

[23] Ministry of Social Affairs and Health. Health, social services and regional government reform in Finland. 2021b. Accessed August 24, 2021. http://alueuudistus.fi/frontpage

[24] Statistics Finland. Wellbeing services counties and the City of Helsinki and Åland. 2025. Accessed February 10, 2025. https://stat.fi/fi/luokitukset/hyvinvointialue/hyvinvointialue_1_20250101

[25] Awanic Ltd. HaiPro: reporting system for safety incidents in health care organizations. 2025. Accessed February 7, 2025. http://awanic.com/haipro/eng/

[26] Mäklin S Kokko P. Health and social care unit costs in Finland in 2017. Finnish Institute for Health and Welfare; 2020. Accessed November 21, 2023. https://www.julkari.fi/bitstream/handle/10024/142882/URN_ISBN_978-952-343-493-6.pdf

[27] Official Statistics of Finland (OSF). Consumer Price Index [online publication]. 2024. Accessed May 23, 2024. Statistics Finland. https://stat.fi/en/statistics/khi

[28] Laatikainen O. Medication-related adverse events in healthcare. Dosis. 2020;36(2):216-229.

[29] Hodkinson A Tyler N Ashcroft DM , et al. Preventable medication harm across health care settings: a systematic review and meta-analysis. BMC Med. 2020;18(1):313. 33153451 10.1186/s12916-020-01774-9 PMC7646069

[30] Mutair AA Alhumaid S Shamsan A , et al. The effective strategies to avoid medication errors and improving reporting systems. Medicines (Basel). 2021;8(9):116. 10.3390/medicines8090046 PMC8468915 34564088

[31] Patient Insurance Centre. Patient safety glossary. 2024. Accessed March 12, 2024. https://www.pvk.fi/terveydenhuolto/potilasturvallisuus/potilasturvallisuussanasto/

[32] European Commission. Costs of unsafe care and cost-effectiveness of patient safety programmes. 2016. Accessed February 17, 2025. https://health.ec.europa.eu/system/files/2017-02/2016_costs_psp_en_0.pdf

[33] Griffey RT Schneider RM Todorov AA. Near-miss events detected using the emergency department trigger tool. J Patient Saf. 2023;19(2):59-66. 36715980 10.1097/PTS.0000000000001092 PMC9974611

[34] Braiki R Douville F Gagnon MP. Factors influencing the reporting of medication errors and near misses among nurses: a systematic mixed methods review. Int J Nurs Pract. 2024;30(6):e13299. 10.1111/ijn.13299 PMC11608931 39225448

[35] Noureldin M Noureldin MA. Reporting frequency of three near-miss error types among hospital pharmacists and associations with hospital pharmacists’ perceptions of their work environment. Res Social Adm Pharm. 2021;17(2):381-387. 32247681 10.1016/j.sapharm.2020.03.008

[36] Kang HJ Park H Oh JM Lee EK. Perception of reporting medication errors including near-misses among Korean hospital pharmacists. Medicine (Baltimore). 2017;96(39):e7795. 10.1097/MD.0000000000007795 PMC5626254 28953611

[37] Naseralallah L Stewart D Price M Paudyal V. Prevalence, contributing factors, and interventions to reduce medication errors in outpatient and ambulatory settings: a systematic review. Int J Clin Pharm. 2023;45(6):1359-1377. 37682400 10.1007/s11096-023-01626-5 PMC10682158

[38] Al Rowily A Jalal Z Price MJ , et al. Prevalence, contributory factors and severity of medication errors associated with direct-acting oral anticoagulants in adult patients: a systematic review and meta-analysis. Eur J Clin Pharmacol. 2022;78(4):623-645. 34935068 10.1007/s00228-021-03212-y PMC8926953

[39] Nolan TW. System changes to improve patient safety. BMJ. 2000;320(7237):771-773. 10720364 10.1136/bmj.320.7237.771 PMC1117771

[40] Valkonen V Haatainen K Saano S Tiihonen M. Improvement proposals and actions in medication error reports: quality and strength: a cross-sectional study. Health Sci Rep. 2024;7(9):e70077. 10.1002/hsr2.70077 PMC11409192 39296637

[41] Reason J. Human error: models and management. BMJ. 2000;320(7237):768-770. 10720363 10.1136/bmj.320.7237.768 PMC1117770

